# 

**Published:** 1840-07

**Authors:** 


### DENTOLOGIA.

We have been asked, by perhaps, some twenty or thirty of our professional brethren, since the notice which we gave in a preceding number, of the above admirable Poem, where it could be had; and on being informed that it had originally been published only for private distribution, and that the whole edition had been disposed of, expressed great regret. They were, therefore, highly gratified at the announcement in the last number of our journal, that the authors, Messrs. Solyman Brown, and Eleazar Parmly, had consented to its re-publication in this periodical; and that it would be commenced in its next issue. This, as will be perceived, has been done, and our subscribers will soon be possessed of a work that should adorn the library or centre table of every member of the profession. The beauty of its composition, and the information which it imparts, cannot fail to please and interest every description of readers. No recommendation of ours, therefore, is necessary to ensure for it a cordial reception and an attentive perusal.——BALT. ED.

### THE AMERICAN JOURNAL OF MEDICAL SCIENCES,

*No. XL VIII.—August*, 1839.

To this publication, the literature of medicine is indebted for many of its richest and most valuable contributions. It has now been in existence twelve years, during which time it has sustained a reputation inferior to that of no similar publication. Besides having for its editor a man, distinguished alike for talents and professional skill, it numbers among its collaborators, many of the most eminent medical gentlemen of the country. Being thus sustained, it at once commends itself to the practitioners of medicine and surgery in all their various departments. We shall enrich our own pages with occasional extracts from it,—one of which—a " case of exostosis of the upper jaw," will be found in another part of this work.—BALT. ED.

### TO OUR PATRONS.

The present number completes half of the first Volume of this Journal, and the publishing committee, are compelled to solicit of all advertising friends, subscribers, agents, and special patrons to forward their dues to the Treasurer, Mr. Jahial Parmly, No. 3 Bond Street, New-York, without further delay.

The expenses of publication must be punctually met as the successive numbers issue from the press, and the committee confidently rely on the patrons of the work for the supply of the necessary means. The difficulty of transmitting funds from the remote sections of the country, the committee are aware, is very great, but to pay the printer without the necessary resources, is still more embarrassing.

SOLYMAN BROWN.

---

Inasmuch as a sufficient number of Subscribers for single copies, or for duplicates of this Journal, did not proffer their support in time to issue a second number during the month of July, the following persons have volunteered their aid by subscribing for twenty copies or more.

If any individuals are disposed to add their names to this catalogue with a view to aid in the circulation of the Journal, they are requested to express their intention to the Publishing Committee, before the appearance of the next number.

Subscribers for less than 20 copies will not be inserted on this page.

### SPECIAL PATRONS:

| | | | |
|---|---|---|---|
| ELEAZAR PARMLY, *Dentist, N. York*, - - - | 40 | copies, | $100 |
| CHAPIN A. HARRIS, M. D. *Dentist, Baltimore* - | 40 | " | 100 |
| GEORGE KEARSING, *Goldbeater, N. York*, - - | 20 | " | 50 |
| E. NOYES, *Dentist, Baltimore*, - - - - - - - | 20 | " | 50 |
| LEVI S. PARMLY, *Dentist, New Orleans*, - - - | 20 | " | 50 |
| W. N. McKENNEY, *Dentist, Fredericksburg, Va.* - | 20 | " | 50 |
| JOHN BURDELL, *Dentist, New-York*, - - - | 20 | " | 50 |
| WALKER & JONES, *Goldbeaters, New-York*, - | 20 | " | 50 |
| SAM'L W. STOCKTON, *Dentist, Phila.* - - - | 20 | " | 50 |
| JAHIAL PARMLY, *Dentist, New-York*, - - - | 20 | " | 50 |
| E. BAKER, *Dentist, New-York*, - - - - - - | 20 | " | 50 |
| A. WOODRUFF BROWN, *Dentist, New-York*, - | 20 | " | 50 |
| EDW'D MAYNARD, *Dentist, Washington City*, - | 20 | " | 50 |
| LEWIS ROPER, M. D. *Dentist, Philadelphia*, - | 20 | " | 50 |
| M. K. BRIDGES, *Dentist, Brooklyn*, - - - - - | 20 | " | 50 |
| S. L. STRINGFELLOW, *Dentist, Baltimore*, - - | 20 | " | 50 |
| J. A. FRAETAS, *Printer, New-York*, - - - | 20 | " | 50 |

---

### TO CONTRIBUTORS.

The Publishing Committee have received several Communications for the pages of the Journal, which, for want of room, they are compelled to reserve for the next number. Well written articles will be thankfully received from all parts of the country, especially if they discuss subjects of practical utility to the dental student.

AGENTS FOR THE

AMERICAN JOURNAL OF DENTAL SCIENCE.

Doct. W. N McKenney, *Fredericksburg, Va.*

F. B. Chewning, *Richmond, Va.*

A. Pierce, *Sussex Court House, Va.*

Chas S. Pleasants, *Painesville, Ohio.*

David Walker & Asahel Jones, 174 *Fulton Street, New York.*

Edward Taylor *Maysville K. Y.*

O. P Laird, M. D., *Columbus, Ga.*

John D. Chevalier, 135 *Fulton st. N. Y.*

Geo Washington Parmly, *N. Orleans.*

A. W. Brown, *New-York.*

F. M. Boykin, M. D. *Smithfield, Va.*

G McDonald, M. D. *Macon, Geo.*

E. Maynard, M. D. *Washington City*

McAllister & McNaughton, *Albany*

M. K. Bridges, *Brooklyn.*

Blanding & Avery, *Columbia, S. C.*

Eleazer Gidney, *England.*

S. Wheeler, M. D *Murfreesboro, N. C.*

W. H. H. Davis, M. D. *Cassville, Oneida Co. New-York.*

B. A. Rodriguez, M. D. *Charles. S. C.*

Ferguson & Goddard, *Louisville, Ky.*

L. S. Stringfellow, *Baltimore.*

J. P. Norman, *Little Rock, Arkansas*

E. E. Crowfoot, *Middletown, Conn.*

Samuel Highley, *Fleet-st. London.*

